# Supplementary material for: [60]Fullerene for Medicinal Purposes, A Purity Criterion towards Regulatory Considerations
Source: Materials (Basel). 2019 Aug 12;12(16):2571. doi: 10.3390/ma12162571 (PMC6719231; doi:10.3390/ma12162571)
Supplement: Supplementary file 1 [file materials-12-02571-s001.pdf]

materials

[60]Fullerene for medicinal purposes, a purity criterion towards regulatory considerations

Sanaz Keykhosravi [1], Ivo B. Rietveld [2], Diana Couto [1], Josep Lluis Tamarit [3], Maria Barrio [3], René Céolin [1] and Fathi Moussa [1,*]

Supplementary Materials

Nanoparticle News April 2004

COMMENTARY

Confusion in the Mass Media

A study linking a fullerene derivative to brain damage in fish made national and international headlines last month, with coverage in a number of major newspapers. Unfortunately, many of these high profile reports misinterpreted the science, generalized the results, or reported the work outside the context of what is currently known about fullerene and nanoparticle toxicity. Provocative headlines turned this single, small study into a poster child for the alleged dangers of fullerenes and nanotechnology.

A major U.S. newspaper, for example, carried the headline "Nanotechnology Linked to Organ Damage – Study" and stated that "the first study to look at the health effects of microscopic, manufactured 'nanoparticles' on aquatic animals has found troubling evidence that the molecules — which scientists are starting to make for research and industry — can trigger organ damage and other toxic effects." Another metropolitan newspaper reported that "buckyballs, discovered two decades ago by a Rice University scientist, cause brain damage in fish" and that a Japanese company "is already making hundreds of tons of the material for industrial purposes..."

Among the problems with these and other media reports are that molecules are confused with nanoparticles, research materials are confused with high-volume commercial products, and only one side of the toxicity story is presented.

Let's take a look at the research, and the facts.

Fullerenes, also called buckyballs or C60, are soccer-ball shaped molecules of carbon. In the recent study, nine juvenile largemouth bass exposed to a water-soluble fullerene derivative at a dose of 0.5 parts per million suffered severe brain damage as measured by lipid peroxidation, or the breakdown of lipids, as shown by laboratory analysis of brain tissue samples. The brain damage seen in the fish exposed to the water-soluble fullerenes was severe: 17 times higher than that seen in nine unexposed animals, according to Eva Oberdoerster, a lecturer with Southern Methodist University who led the study.

The work is significant in that it raises a red flag about the possible environmental impact of the particular fullerene derivative under investigation. Using a solubilization procedure, the researchers formulated C60 into a water-soluble, or hydrated, fullerene nanoparticle of about 30 to 100 nm in size.

"Fullerenes don't have any water solubility," says Felipe Chibante, president of BuckyUSA, a Houston, TX-based producer of fullerenes. "[The researchers] altered it to make a water-soluble derivative, and derivatives are whole different ballgame."

The fullerene derivatives produced by Oberdoerster are not chemically or structural equivalent to buckyballs, although the media tended to blur this distinction.

"They're promoting this as if they tested fullerene-C60 – and it's completely misleading. This work isn't relevant to anyone making fullerenes, unless they're making nanoparticles out of them. Nanoparticles and molecules aren't the same thing," says Stephen R. Wilson, a chemistry professor at New York University and chief scientific officer of C Sixty, Inc.

Furthermore, these fullerene derivative nanoparticles are not mass-produced materials, as some newspapers reported. "What we're doing in our lab with [fullerene-C60] molecules in solution is completely different from hydrated fullerene nanocrystals, and that's completely different from what's coming out of a 40-tons-per-year plant," says Wilson.

"We normally ship micron-size powders," says Chibante of the fullerenes produced by his company. "You get almost nothing through a 250 mesh." (Particles smaller than 50-60 microns would pass through a 250 mesh sieve.)

Wilson points out that a number of studies have previously demonstrated "the complete lack of toxicity of C60 itself." For example, in a study in which mice were injected with micronized C60 [F. Moussa, et al., Fullerene Sci. Techn. 3, 333 (1995)], French researchers found neither a "lethal effect nor acute toxicity." In a further study lasting eight weeks in duration, the researchers reported that all of the mice were still alive without any behavioral trouble at the conclusion of the study, and that "C60 has no subacute toxic effect."

Not only has C60 been shown to be non-toxic, but various derivatives have proven to be beneficial to human health. "There are thousands of possible fullerene derivatives, and some of them have very good biological properties," says Chibante.

In fact, Wilson's company, C Sixty Inc., has developed several fullerene-based drug candidates that are now in preclinical trials. One is under investigation for dermatological and cosmetic applications to minimize skin aging due to sun exposure, and another is being developed to fight Parkinson's and other diseases.

Quite simply, the message here is that the results of a single study of fullerene derivative nanoparticles should not be used to make blanket statements about buckyballs or the field of nanotechnology in general. A fullerene derivative is not the same as a fullerene. A molecule isn't a nanoparticle. Does it take a PhD to see the distinction?

Mindy N. Rittner
Editor

Figure S1. Commentary by M.N. Rittner about the C60 toxicity.

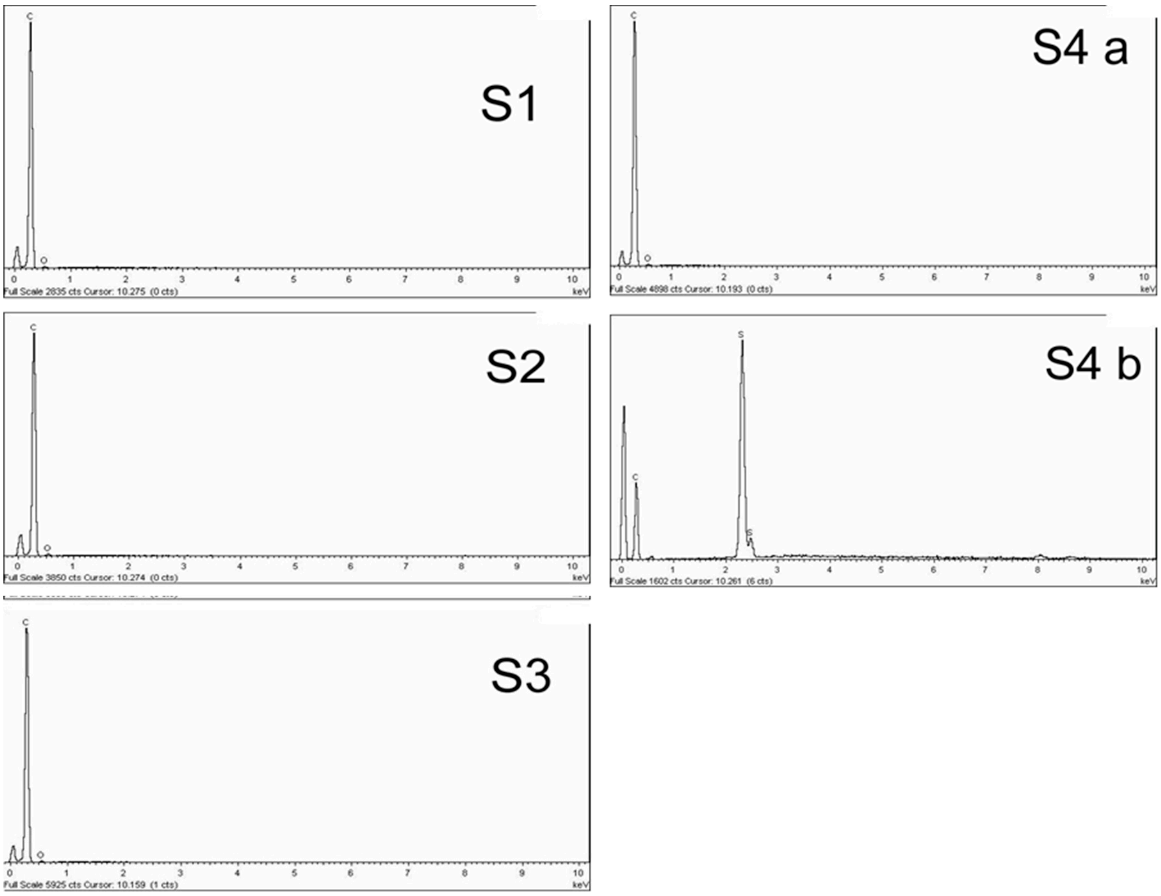

Figure S2. Energy dispersive spectroscopy of the C60 samples S1–S4. S4b shows the presence of sulfur in some of the particles present in sample S4.

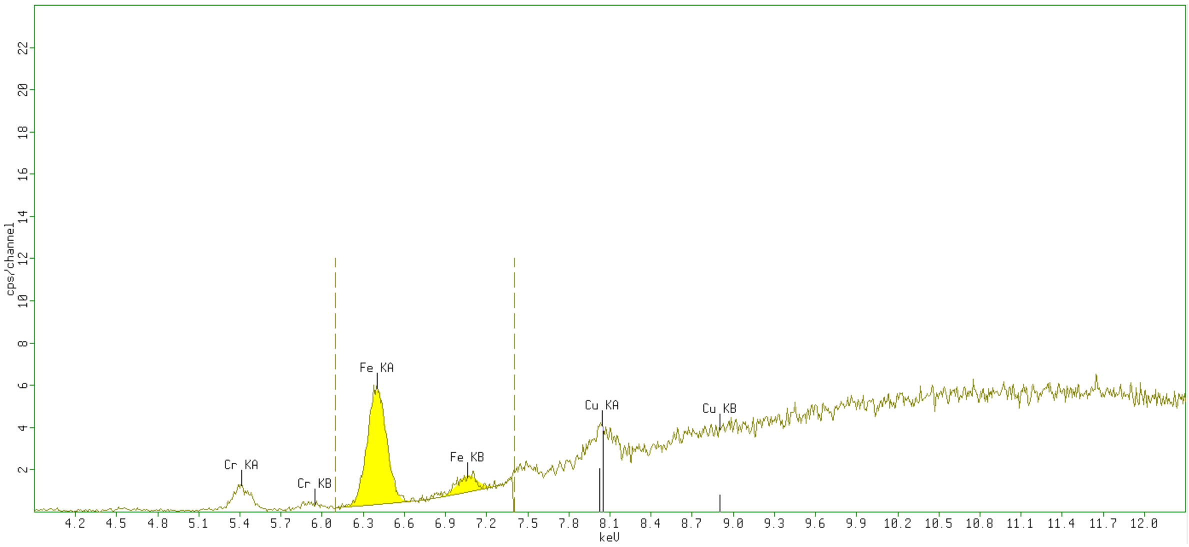

Figure S3. Energy dispersive fluorescence showing the presence of iron and traces of chromium and copper.
